# Response to "The Glasgow prognostic score is unsuitable for stroke prediction in infective endocarditis"

**DOI:** 10.1590/1806-9282.20241531

**Published:** 2025-05-02

**Authors:** Cihan Aydın, Aykut Demirkıran

**Affiliations:** 1 Tekirdağ Namık Kemal University, Faculty of Medicine, Department of Cardiology – Tekirdağ, Türkiye.

Dear Editor,

We thank Josef for his interest in our article. We want to respond to your criticism and contributions. After pneumonia, intra-abdominal abscess, and sepsis, infective endocarditis is the most common infectious condition that causes a life-threatening risk and is linked to higher rates of mortality and morbidity^1^. It was stated by Mr. Finsterer that there was no difference between Glasgow prognostic score (GPS) levels. However, in our study, GPS was found to be higher in Group 1 who had a stroke [Group 1=2(1–2) vs. Group 2=1(0–2), p<0.001]^2^. As is known, many biomarkers such as C-reactive protein and albumin can increase for various reasons^3^. In addition, many variables that suppress inflammation may cause lower detection of these biomarkers. We mentioned these issues in the limitation section of our study. In our study, we tried to create two homogeneous groups in terms of diseases, such as diabetes, heart failure, kidney failure, and liver failure. As a second criticism, it was mentioned that complications of infective endocarditis may cause GPS elevation. However, the GPS values of the patients were calculated at the time of admission to the hospital before the complications developed.

Scores should not limit us, of course. However, they can be practical in making decisions on some issues and are widely recommended in many guidelines. Also, no score is perfect and has shortcomings. There are of course many conditions that can cause a stroke. In our study, patients with carotid artery stenosis were excluded. We could have added in the limitation section that the coagulopathy panel could not be examined.

As we mentioned in the article, stroke diagnosis of patients is made using physical examination, computed tomography, and cranial magnetic resonance imaging methods.

Although scoring systems such as GPS cannot replace physical examination, anamnesis, and other diagnostic methods, we believe that they can help us in diagnosis and treatment.
